# Seasonal climatic variability shapes immune responses and infection risks in the common bluetail damselfly

**DOI:** 10.1007/s00442-026-05882-w

**Published:** 2026-03-24

**Authors:** Shatabdi Paul, Md Tangigul Haque, Marie E. Herberstein, Md Kawsar Khan

**Affiliations:** 1 School of Natural Sciences, Macquarie University, Sydney, NSW-2109 Australia; 2 Department of Biology, Chemistry and Pharmacy, Freie Universität Berlin, Berlin, Germany; 3 Leibniz Institute for the Analysis of Biodiversity Change, Bonn, Germany; 4 Department of Biology, University of Hamburg, Hamburg, Germany; 5 Applied BioSciences, Macquarie University, Sydney, NSW-2109 Australia; 6 Department of Biochemistry and Molecular Biology, Primeasia University, Dhaka, Bangladesh

**Keywords:** Anthropogenic climate change, Parasitism, Encapsulation, Host–pathogen interaction, Insect immunity, Seasonality

## Abstract

Understanding how a changing climate influences host-parasite interactions is important to predict disease-driven extinction risks. Insect immune responses are sensitive to seasonal climatic factors such as temperature, humidity, and rainfall, however, the influence of seasonal climatic fluctuations on insect immune responses and parasite prevalence remains poorly understood. To address this gap, we studied seasonal variation in immune response and endoparasite (protozoan gregarine) prevalence (proportion of infection) in *Ischnura heterosticta* damselflies. Damselflies may experience increased food availability during warmer seasons, providing greater energetic resources for the metabolically costly synthesis of melanin a key component of the prophenoloxidase immune response; therefore, we predicted higher melanisation and lower parasite (gregarine) prevalence in warmer than cooler months. In accordance with our prediction, we found stronger melanisation and lower gregarine prevalence in summer. We further found that melanisation increased with air temperature and decreased with rainfall and humidity while gregarine prevalence decreased with air temperature and increased with humidity and rainfall in females but not in males. Our study provided evidence that natural seasonal variation in climate can impact patterns of immune response, host-parasite interactions and infection prevalence across seasons. While short-term warming during favourable seasons may enhance host immune response, long-term or extreme climate change might disrupt host-parasite relationship by altering resource availability, humidity patterns, or insect thermal limits, thereby contributing to seasonal declines in host populations.

**Supplementary Information:**

The online version contains supplementary material available at 10.1007/s00442-026-05882-w.

## Introduction

Seasonal fluctuation in climate may contribute to the decline of insects worldwide by increasing disease prevalence and spread (see Harvey et al. 2020). Insect immune responses are influenced by factors such as temperature, humidity, and rainfall (Martin and Hillyer 2024) and hence climate change-driven increases in temperature is predicted to impact insect immune responses and consequently disease prevalence across a spatiotemporal scale (Paul et al. 2024; Reece et al. 2017). Seasonal climate can alter prevalence, intensity of parasitism, and the fitness costs imposed by parasites, thus, estimating the impact parasitism and the cost of parasitism across seasons can help determining the risk of local population decline across seasons (see Paul et al. 2024).

Climatic factors such as temperature, rainfall, and humidity impact various aspects of insect life history traits, including the immune response (Cohen et al. 2020; da Silva et al. 2021; LoScerbo et al. 2020; Mlynarek et al. 2015). Higher temperatures can increase metabolic costs and energy required to perform basal physiological functions, thereby reducing resources available for immune responses, with the potential of increasing parasitism or diseases in warmer months (Khan and Rolff 2025). For example, damselfly larvae (*Coenagrion puella*) exposed to simulated heat waves under laboratory conditions with ad libitum feeding showed reduced energy reserves and lowered immunity, indicating that direct heat stress, rather than limited resource availability, was responsible for these effects (Tüzün and Stoks 2021). On the other hand, higher temperatures can increase the availability of food and nutrition, which can increase resources for immune response, thereby reducing parasitism or diseases in warmer months. For example, stronger immune responses were detected under higher temperatures in mosquitoes (see Murdock et al. 2012) and sepsid flies (Gourgoulianni et al. 2023). Similarly, in *Lestes forcipatus* and *Ischnura elegans* damselflies, stronger immune responses were observed in warmer seasons (Robb and Forbes 2005; Raczyński et al. 2022). The impact of temperature, therefore, could increase or decrease immune responses and consequently the prevalence and intensity of parasitism, with the direction and extent depending on the host-parasite system and local climatic conditions.

Insect immune responses include cellular and humoral components, with melanisation playing a major role in humoral defence against pathogens. Melanin in insects is a natural pigment that determines colouration and can provide protection against environmental stressors. Melanin occurs mainly as two types: eumelanin and pheomelanin, which are responsible for black, brown, red, and yellow coloration. The prophenoloxidase (proPO) cascade drives melanin production, encapsulating pathogens and removing parasites from the insect body (see Khan and Rolff 2025; Ilvonen et al. 2018; Siva–Jothy 2000). Environmental factors affect immune responses, measured as PO activity or degree of melanisation, thereby modify infection rates in insects (Carter et al. 2021; Ismail et al. 2024; Scharsack and Franke 2022). However, little is known about the impact of climate fluctuations on insect immune responses and seasonal parasite prevalence, despite their impact on disease risk and the potential for seasonal population declines under a changing climate.

Here, we aim to understand the pattern of immune response and parasite prevalence across seasons and determine the underlying climatic drivers. We used the Australian common bluetail damselfly (*Ischnura heterosticta*) and an endoparasitic gregarine (Apicomplexa: Protozoa) as a host-parasite study system. We determined melanisation as an index of immune response and measured gregarine prevalence across seasons. We hypothesised that immune response and parasitism would vary with temperature across seasons. Specifically, (1) higher temperatures in warmer months may increase the immune response and reduce parasitism if thermal conditions promote physiological activity and development; alternatively, (2) parasitism may increase in warmer months if high temperatures reduce host immune investment and/or favour parasite development.

## Methods and materials

### Host-parasite study system

Damselflies are semi-aquatic insects and hosts to endoparasitic gregarines (Zawal and Dyatlova 2008), which are transmitted by drinking water contaminated with gregarine oocysts, or through ingesting contaminated prey such as flies (Hecker et al. 2002). In the damselfly gut, gregarine oocysts develop into sporozoites, which attach to the damselfly posterior gut epithelium, then transform into mature trophozoites, ultimately developing into reproductive gametocysts (Baker 2023). Gregarines may damage the damselfly gut lining and reduce damselfly fitness, impacting their survival and lowering egg production in females (Cordoba-Aguilar and Munguía-Steyer 2013; Kaunisto et al. 2017).

**Fig. 1:**
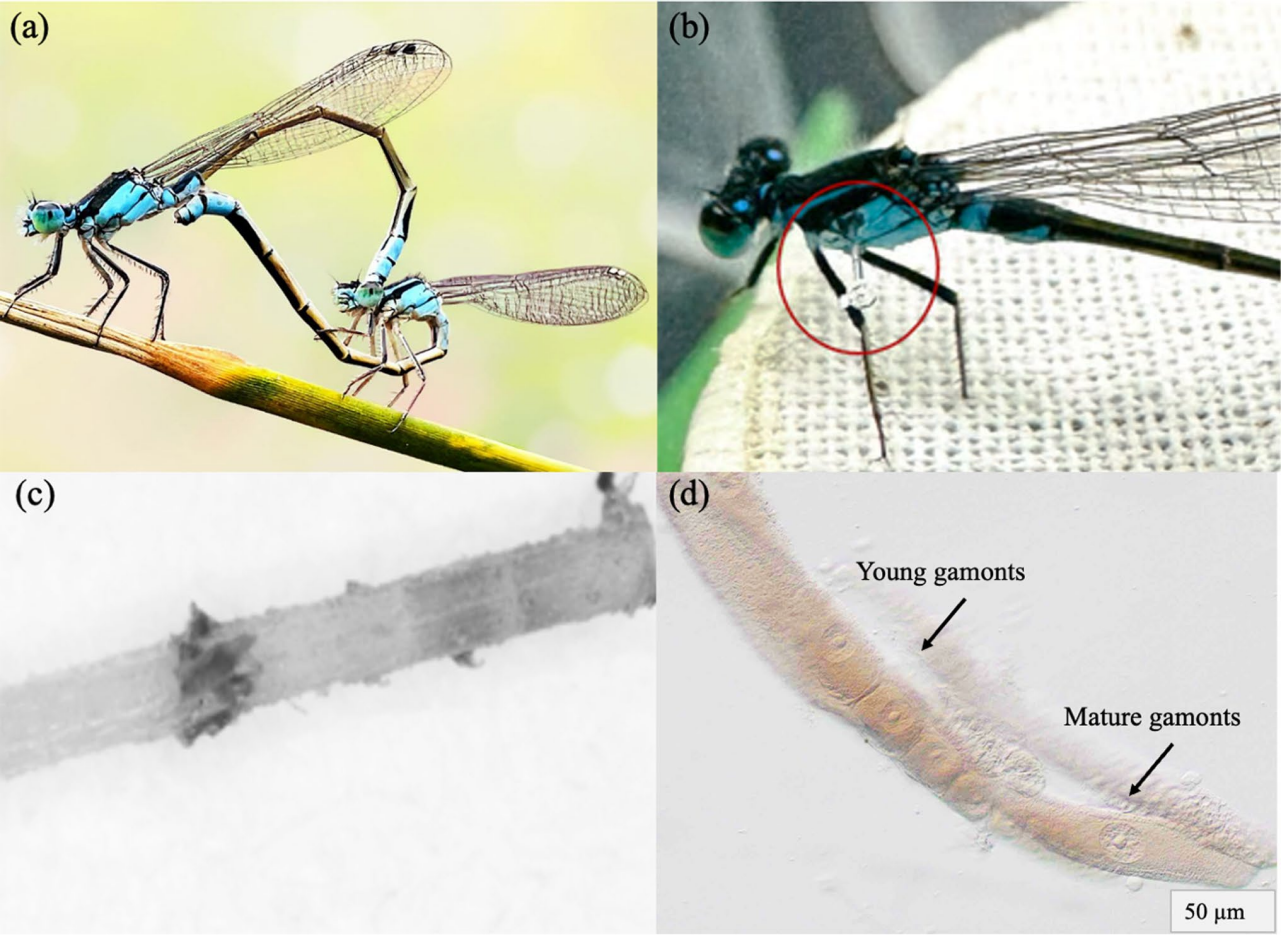
Photograph (**a**), showing a mating pair of *Ischnura heterosticta* damselfly (male colour blue and immature female mimic male colour); Photograph (**b**), showing a male damselfly with a nylon filament inserted; (**c**) 8 bit microscopic image of a filament after ~ 24 h of experiment removed from a female damselfly; (**d**) an image of endoparasite gregarine (associative form of young gamonts and mature gamonts) (Clopton and Hays, 2006). Image credit © S. Paul

We studied seasonal variation in immune response and gregarine parasite prevalence in *Ischnura heterosticta* damselflies, a medium sized (body length: 33.7 ± 0.08 mm) damselfly belonging to the Coenagrionidae family (Haque et al. 2025). In the field, male *I. heterosticta* are distinguished by a black and blue head and thorax, and a black abdomen with blue bands (Fig. 1a). *Immature I. heterosticta* females initially resemble males in colour and turn grey as they mature (Fig. 1a). This species is widely distributed throughout Australia and found in lentic and lotic habitats which are naturally parasitised by *Arrenurus* water mites and protozoan gregarines (Paul et al. 2022; 2024).

### Study site

We collected damselflies from a natural population located on the Wallumattagal campus of Macquarie University, NSW, Australia. The study site is a small artificial lake with an area of approximately 895 m^2^ and a perimeter of 212 m. The lake is permanent with stagnant water flow. We surveyed the study site every month from March 2024 to February 2025, covering the entire flight season of this species. No permits were required for damselflies collection from the site, as this species is not a protected species and the field site is not part of a national park or protected area.

### Determining immune response

We captured damselflies (males, mature and immature females) from the field with insect-catching nets (dimensions: 1260 mm handle, 456 mm diameter hoop, 81 cm long net bag) while walking along the edge of the water body and adjacent grasslands. For each sampling day, we covered the same study area and spent approximately 30 min collecting 35–40 damselflies (14 sampling days collecting 10 control and 30–35 experimental damselflies each day for a total n = 470). We transported the damselflies to the laboratory within five minutes of capture and placed them into a plant growth chamber (Plant growth facility, Macquarie University, NSW, Australia) for acclimation for two hours. We set the temperature at 25 °C, the humidity at 80% and the light: dark cycle at 16:8 h.

We quantify the immune response in damselflies using the encapsulation response assay (Nagel et al. 2011). We used a needle holder to insert a sterile 3 mm (filament insertion depth was 2 mm) long nylon monofilament (diameter 0.20 mm; treated with fine sandpaper) into the body of the experimental damselflies (Koskimäki et al. 2004; Nagel et al. 2011; Rantala et al. 2000). We insert the nylon filament into the thorax below the lateral stripe (Fig. 1b). We maintained the consistency of the length of inserted filaments throughout the experimental procedures. Control animals were not manipulated. We kept all damselflies individually in a plastic drinking cup (100 ml) covered with a cotton mesh and a wood dowel for perching. We placed the cups in the growth chamber for 24 h (based on studies that showed that the melanisation reaction occurred within 24 h after implanting nylon filaments; Galko and Krasnow 2004; Tang 2009). Melanin from the damselfly hemolymph binds naturally to the nylon surface as part of the encapsulation response. After 24 h, we recorded the survival status of control and experimental animals (dead, or alive). We removed the nylon from the experimental animals with the needle holder and placed it in ethanol (70%) in an Eppendorf tube (0.5 ml). Damselflies were euthanised at − 30 °C and randomly selected experimental individuals (n _total_ = 140) from each sampling day were dissected to determine the presence of gregarines.

Overall, mortality at 24 h was high (94.89%) but did not differ between the nylon treatment and the control. Therefore, the observed melanisation is likely to reflect baseline immune capacity and short-term stress responses induced by captivity.

We photographed the nylon filaments at 3.2 × with an OLYMPUS SZX16 stereo microscope under standard lighting using OLYMPUS cellSens imaging software. We took the filament images from three different angles and used ImageJ software to calculate the amount of melanin present on the filament. We measured melanisation by quantifying the proportion of the inserted nylon filament surface that was covered with dark pigment. This pigment binds to the filament as part of the encapsulation immune response and was quantified as a greyscale value (average darkness). We also measured the greyscale value of the part of the filament that remained outside of the damselfly thorax to control for colour variation in the nylon filament. All measurements were identical, indicating consistent colour conditions across images. In ImageJ, we converted the RGB image to 8-bit greyscale and considered 0 as pure black and 255 as pure white (the lower greyscale value indicated a higher amount of melanin present on the filament). We took the average of greyscale values from three images and subtracted it from 255 (called reverse greyscale value) to make it easier to interpret (lower value, lower melanisation) (Ferguson and Sinclair 2017). We collected monthly maximum and minimum temperature data for 2024–2025 from the Bureau of Meteorology (BOM: http://www.bom.gov.au/climate/data/index.shtml) and calculated monthly average temperature (°C), rainfall (mm), and humidity (%) for each month that we surveyed. The average autumn, spring, and summer temperatures of this study area during our collection period were 19.83 ± 1.67 °C, 19.28 ± 2.61 °C, and 24.06 ± 0.48 °C, respectively. Average autumn, spring, and summer rainfall levels were 132.84 ± 62.96 mm, 44.54 ± 9.79 mm, 66.84 ± 37.82 mm, respectively. Average autumn, spring, and summer humidity was 60.70 ± 14.15%, 53.49 ± 9.16% and 60.1 ± 5.81%, respectively.

### Statistical analyses

We applied the DurgaDiff function of the *Durga* R package to determine mean differences of reverse greyscale value (as a measure of melanisation) and gregarine prevalence between sexes, between female developmental stages (please see the supplementary information) and across seasons (Khan and McLean 2024). This function calculated 95% confidence intervals of the mean difference by bootstrapping 1000 times. We applied a generalized linear model (GLM) to identify the effect of climatic factors (monthly average temperature, rainfall, and humidity) on melanisation and gregarine prevalence. We fitted the GLM models with melanisation/gregarine prevalence as the response variables, and climatic factors as fixed effects. We analysed all data in R version 4.0.3 (R Core Team 2020) using packages “*lme4*” for fitting and analyzing mixed models (Bates et al. 2014), “*performance*” for checking overdispersion (Lüdecke et al. 2021), and “*Durga*” for estimating and plotting effect sizes (Khan and McLean 2024). All values are estimated ± standard error. For model description, please see the supplementary information.

## Results

### Melanisation and gregarine prevalence between sexes and female developmental stages across seasons

**Fig. 2:**
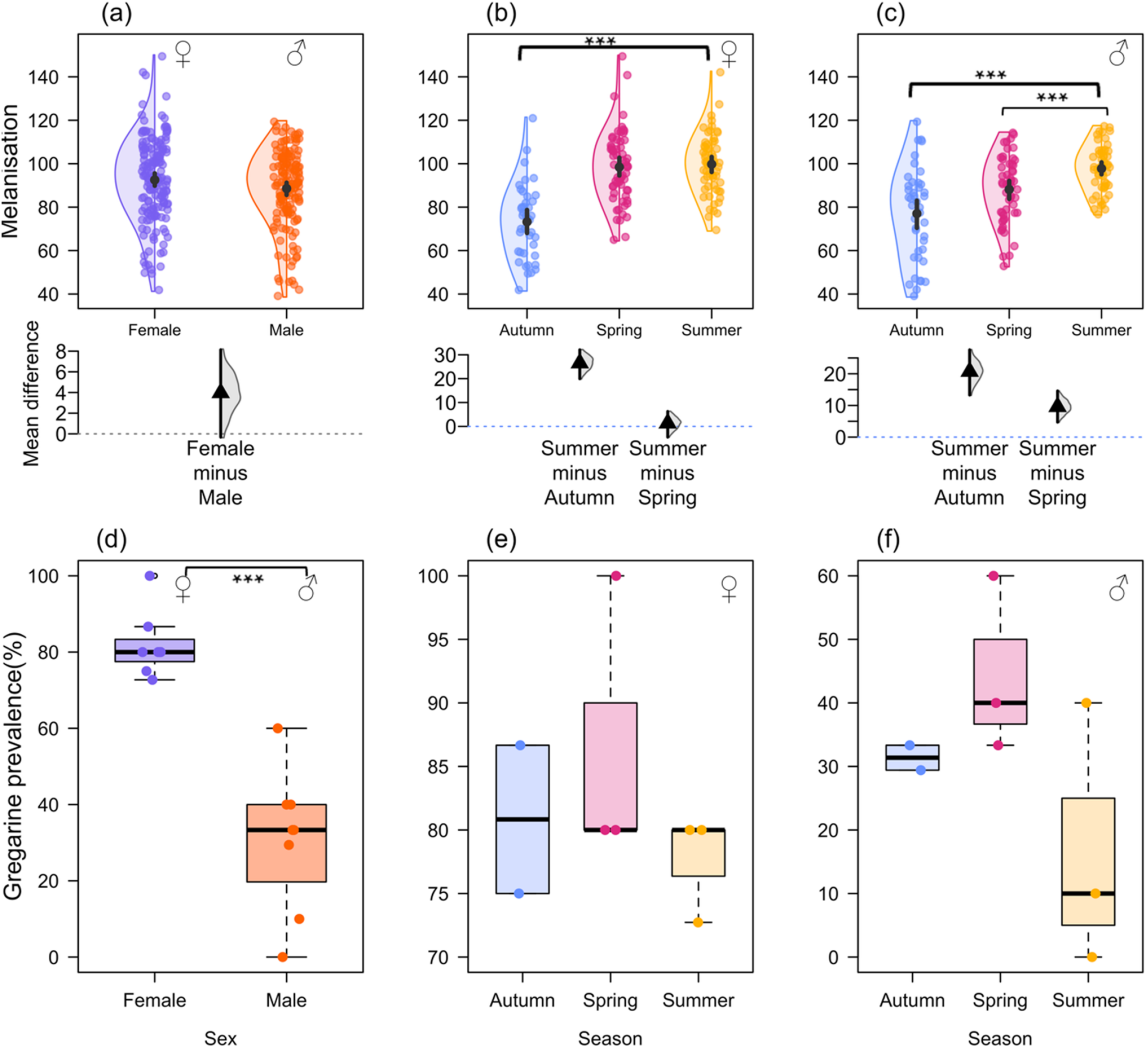
Melanisation response (greyscale value) and gregarine prevalence (%) in *I. heterosticta* damselflies across seasons. Melanisation response and variation in gregarine prevalence (**a**), (**d**) between sexes; across seasons (**b**), (**e**) in females; and (**c**), (**e**) in males. In the upper panel of plots (**a**–**c**), the black circle represents the mean, and the vertical bar represents confidence intervals (CI) of both sexes across seasons. In (**a**), coloured dots represent melanisation, and each coloured circle in (**b**) and (**c**) represents a sampling event across seasons for females and males, respectively. In the lower panel, the triangle represents the mean difference, vertical line represents the 95% CI of the mean difference from 1000 bootstraps. Boxplots (**d**–**f**), showing the difference in gregarine prevalence between sexes and across seasons, where bold lines indicate the median, and bottom and top borders depict the 25th and 75th percentiles. The error bars extend downward from the first quartile to the minimum and upward from the third quartile to the maximum data points. For each sampling event, 35 individuals (n = 35) were tested to evaluate differences in melanisation between sexes and among seasons (Fig. **a**–**c**). However, we used a subset of data (n = 10, randomly selected from each sampling event) to determine the percentage of damselflies infected (Fig. **d**–**e**). *Brackets indicate significant pairwise differences between groups; asterisks denote significance levels (***p < 0.001)

Melanisation differed between mature and immature females (Fig. S3), being higher in mature female, both however, being higher than in males and thus we pooled them for subsequent analyses. Melanisation was higher in female than males (mean difference in reverse greyscale value: 3.96, 95% CI [0.45, 8.19], Fig. 2a). Similarly, gregarine prevalence was higher in mature than immature females, (Fig. S3d) but still higher than in males, and we thus pooled them to compare females with males, with females having a substantially higher prevalence than males (mean difference: 0.54, 95% CI [0.4, 0.66], Fig. 2d). Melanisation was higher in summer (average reverse greyscale value: 98.80 ± 12.43) and spring (93.68 ± 16.66) and lower in autumn (75.15 ± 19.20) for both sexes (Fig. 2b, c; Table 1; also see supplementary for GLM results). Gregarine prevalence was higher during spring (66.66%) and autumn (54.76% than in summer (45.09%) (Fig. 2d, e; Table 1; also see supplementary for GLM results).

**Table 1:** Mean differences showing the variation in melanisation of female and male *I. heterosticta* damselflies across seasons

Sex	Response variable	Group difference	Mean difference	95% CI
Female	Melanisation	Summer–Autumn	26.53	[20.09, 32.22]
		Summer–Spring	1.34	[− 4.5, 6.41]
Male		Summer–Autumn	20.72	[13.61, 27.25]
		Summer–Spring	9.60	[4.48, 14.43]
Female	Gregarine prevalence	Spring–Autumn	0.05	[− 0.06, 0.18]
		Spring–Summer	0.09	[0, 0.2]
Male		Spring–Autumn	0.13	[0.02, 0.26]
		Spring–Summer	0.27	[0.04, 0.46]

### Correlation of melanisation and gregarine prevalence with climatic factors

**Fig. 3:**
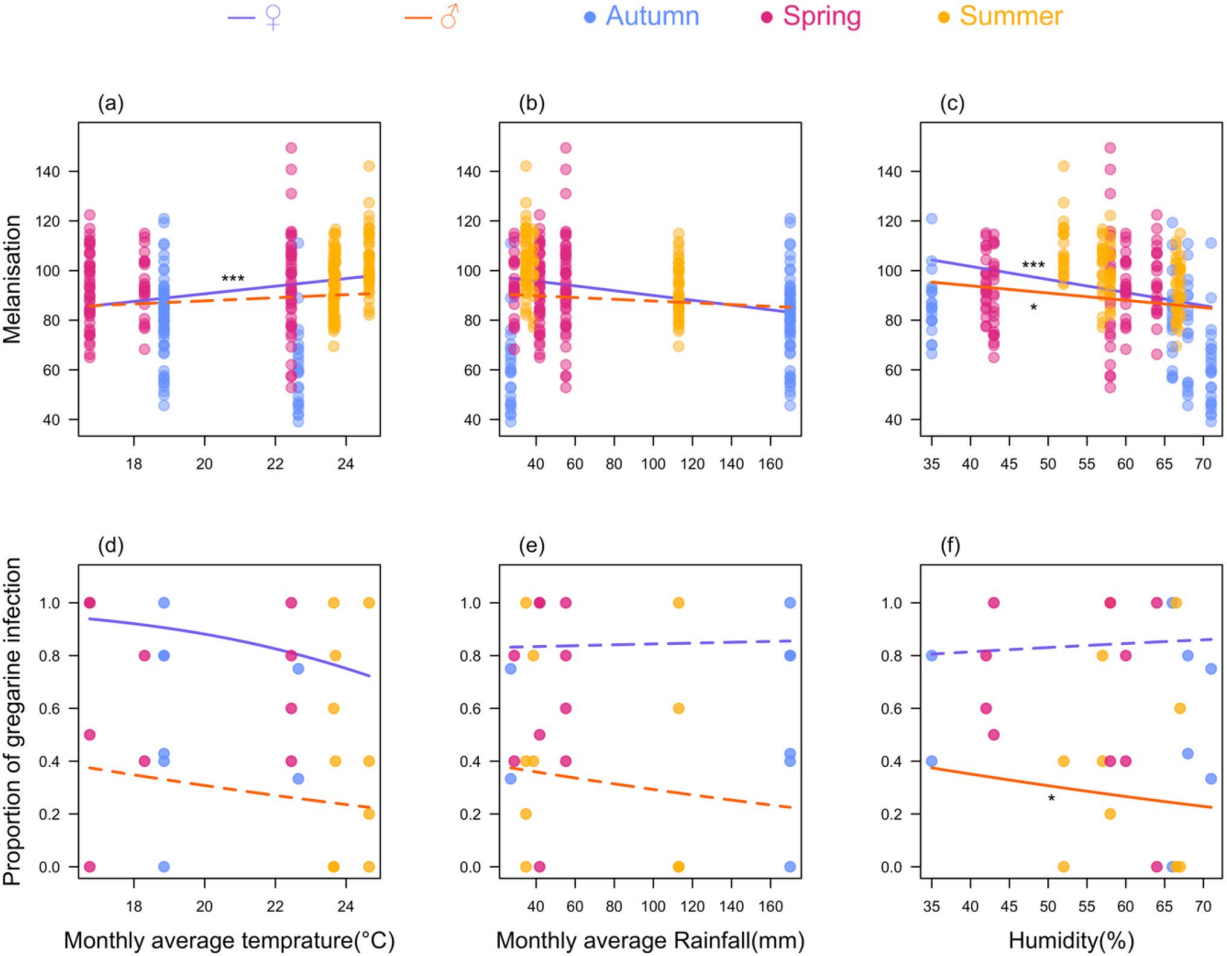
Correlation of melanisation response (greyscale value) and gregarine prevalence (proportion of gregarine infection) in *I. heterosticta* damselflies with climatic factors (monthly average temperature, rainfall, and humidity). Plots (**a**–**c**) and (**d**–**f**) show the correlation of melanisation and gregarine prevalence with climatic factors across three seasons in females and in males, respectively. Each circle represents a sampling event. The fitted lines represent the overall trend of the data points. We used a subset of data (n = 10 from each sampling event) for calculating the effect of climatic factors on gregarine prevalence. Asterisks denote significance levels (*p < 0.05, ***p < 0.0001)

In females, melanisation was positively correlated with monthly average temperature (GLM, estimate = 1.63 ± 0.50, z = 3.25, p = 0.001; Fig. 3a), but negatively correlated with rainfall (GLM, estimate = − 0.07 ± 0.02, z = − 2.67, p = 0.008; Fig. 3b) and humidity (GLM, estimate = − 0.58 ± 0.13, z = − 4.35, p < 0.0001; Fig. 3c). In males, melanisation was also negatively correlated with humidity in (GLM, estimate = − 0.31 ± 0.13, z = − 2.28, p = 0.02; Fig. 3c), but not with temperature and rainfall (Table 2; Fig. 3a, b).

**Table 2:** Correlation of melanisation and gregarine prevalence with monthly average temperature, rainfall, and humidity across seasons in female and male *I. heterosticta* damselflies

Sex	Response variable	Fixed effect	Estimate	Std. Error	z value	P value
Female	Melanisation	Monthly average temperature	− 1.63	0.50	3.25	0.001
		Rainfall	− 0.07	0.02	− 2.67	0.008
		Humidity	− 0.58	0.13	− 4.35	< 0.0001
Male		Monthly average temperature	0.68	0.52	1.31	0.19
		Rainfall	− 0.02	0.02	− 0.92	0.35
		Humidity	− 0.31	0.13	− 2.28	0.02
Female	Gregarine prevalence	Monthly average temperature	− 0.22	0.12	− 1.81	0.07
		Rainfall	0.001	0.006	0.20	0.83
		Humidity	0.01	0.02	0.39	0.69
Male		Monthly average temperature	− 0.09	0.08	− 1.06	0.28
		Rainfall	− 0.003	0.004	− 0.76	0.44
		Humidity	− 0.05	0.02	− 2.37	0.01

While gregarine prevalence was greater in mature than immature females (Fig. S4), climate patterns were similar (Fig. S4) and we pooled the data. In females, gregarine prevalence decreased with monthly average temperatures (GLM, estimate = − 0.22 ± 0.12, z = − 1.81, p = 0.07; Fig. 3d), but not with rainfall (Table 2, Fig. 3e) and humidity (Table 2, Fig. 3f), In males, gregarine prevalence did not correlate with temperature but was negatively correlated with rainfall (Table 2, Fig. 3e) and humidity (GLM, estimate = − 0.05 ± 0.02, z = − 2.37, p = 0.01; Fig. 3f).

## Discussion

We found that females and males had higher melanisation in summer compared to spring and autumn. Melanisation increased with temperature but decreased with rainfall and humidity, whereas gregarine prevalence was higher in females throughout seasons and weakly negatively correlated with temperature. We further found that melanisation was higher in seasons when gregarine prevalence was lower (i.e. summer). Overall, these results suggest that seasonal shifts in melanisation play an important role in modulating infection risk in *I. heterosticta* across the year.

### Relationship between melanisation and gregarine infection

**Fig. 4:**
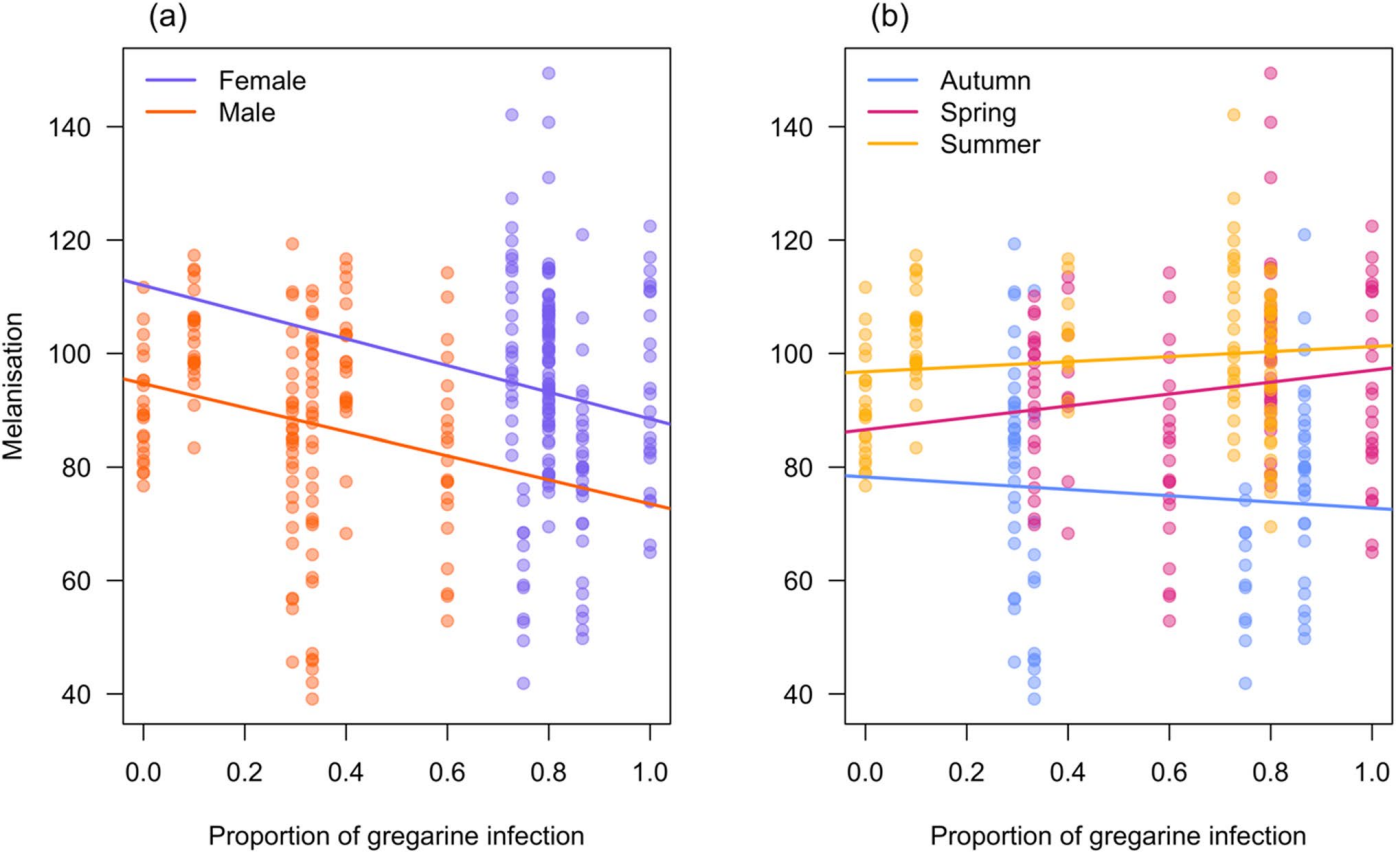
Correlation of melanisation response (greyscale value) and gregarine prevalence (proportion of gregarine infection) in *I. heterosticta* damselflies. Plot (**a**) shows the relationship between individual melanisation response and the proportion of gregarine infection, differentiated by sex (females and males). Plot (**b**) shows the same relationship across seasons (autumn, spring, and summer). Each circle represents an individual damselfly, with gregarine prevalence calculated as the proportion of infected individuals within each sampling event. The fitted lines represent the overall trend of the data points for each group

Our results revealed an inverse relationship between melanisation and infection, pooling the data from all seasons (Fig. 4a, b; also see supplementary information). Similarly, crickets (Fedorka et al. 2013) and dung flies (Gourgoulianni et al. 2023) also showed stronger immune responses in warmer seasons, which correlated with lower infection risks. Warmer conditions enhanced melanin-producing enzyme activity which may aid parasite clearance. Conversely, damselflies had a lower immune response in spring, which may have increased their susceptibility to gregarines, resulting in higher gregarine prevalence. These findings are consistent with broader evidence across taxa, including frogs (Anura), where melanin-based immunity contributes to pathogen resistance (Laumeier et al. 2023), suggesting a potentially conserved role of melanisation in reducing parasite infection. Overall, our results indicate that seasonal shifts in immune responses shaped infection risks in *I. heterosticta* damselflies, but direct experimentation is needed to assert this causality.

### Seasonal variation of melanisation and its correlation with climatic factors

Our study showed that melanisation in *I. heterosticta* damselflies was higher in summer compared to spring and autumn. Consistent with our findings, immune responses in cricket (*Allonemobius socius*) were also higher during warmer months (Fedorka et al. 2013). Insects are likely able to mount a stronger immune response in summer (see Adamo and Lovett 2011), due to greater resource availability, which enables a relatively higher investment in immunity (Hangartner et al. 2013; Kiss et al. 2020; Rivera-Rea et al. 2022). For instance, food sources for damselflies, such as Diptera, Hymenoptera, and Coleoptera, are more abundant during the summer (Lim et al. 2021). Increased food availability enhances nutrition, which in turn boosts immune responses in damselflies and other insects (Leung, B et al. 2001; Kiss et al. 2020). Direct experiments are needed to unlink the effect of temperature from seasonal food availability on host immunity under warming conditions.

We found that temperature, rainfall, and humidity, influenced melanisation, with temperature being positively, and rainfall and humidity negatively correlated with melanisation. Our study supports previous findings, where higher temperatures enhanced melanisation in tropical species or those adapted to warmer climates, as seen in *Sepsis thoracica*, *Galleria mellonella*, and *Sarcophaga africa* (Gourgoulianni et al. 2023; Mastore et al. 2019). Summer temperatures have a positive association with phenol-oxidase (PO) enzyme activity—a key enzyme of melanin production—as shown in the Caribbean termite *Nasutitermes acajutlae* (Fuller et al. 2011) and mealworm larvae *Tenebrio molitor* (Catalán et al. 2012). Similarly, the negative correlation between melanisation and rainfall or humidity was also reported in *Parnassius clodius* butterflies (Zaman et al. 2019). Drier conditions increase the expression of proPO and consequently increase PO activity (e.g. burying beetles: Urbański et al. 2021), but are unlikely to be in relation to a desiccation protection function of melanin pigments in the insect cuticle (e.g. Rajpurohit et al. 2016).

Importantly, our results align with broader interspecific patterns: seasonal variation in cuticular melanisation in odonates suggests a role of thermal melanism across seasons (Novella-Fernandez et al. 2023). Across species, color lightness tends to increase with temperature and decrease with humidity or pathogen load, supporting a link between melanisation and environmental or immune adaptation (Pinkert et al. 2017; Stelbrink et al. 2019; Laumeier et al. 2023). While previous studies often focus on cuticular melanisation, our detailed measurements of hemolymph melanisation across sexes and seasons provide deeper insight into seasonal modulation of immune investment.

### Seasonality of gregarine prevalence and its correlation with climatic factors

We found higher gregarine prevalence in spring compared to autumn and summer. Our study corroborates previous findings of higher parasitism in cooler months, i.e., water mite infection in damselflies (Paul et al. 2024; Robb and Forbes 2005) and endoparasite infections in mosquitoes during cooler periods (Farner et al. 2025, preprint; Trzebny et al. 2024). Lower spring temperature, rainfall, and humidity may drive this seasonal shift in gregarine prevalence in damselflies by influencing gregarine life history traits. Spring conditions increase development, density, and infectivity of the free-living stages of gregarines (Paul et al. 2024; Trzebny et al. 2024), a pattern also observed in other endoparasites such as *Lambornella clarki* in mosquitoes (Ismail et al. 2024) and gregarine *Blabericola migrator* infection in cockroaches (Kolman et al. 2015). While lower rainfall and humidity were linked to higher parasitism in damselflies in our study, the correlation was weak (see also microsporidian occurrence in mosquitoes Trzebny et al. 2024). Wetter conditions may still contribute to increased infection prevalence by increasing parasite abundance due to lower water levels (Shearer and Ezenwa 2020; Trzebny et al. 2024), oocyst viability, transmission, or greater host exposure to parasites. As shown above, seasonal climate may impact host immunity, further influencing the prevalence of gregarine infection.

A limitation of our study is that we did not include host or community population density in our analyses. Density-dependent effects may influence resource availability, immune investment, and parasite transmission, potentially contributing to the observed seasonal variation in melanisation and gregarine prevalence. Incorporating density measures in future studies would help clarify the relative roles of climate, immunity, and transmission dynamics.

## Conclusion

Overall, we showed seasonal dynamics in melanisation and gregarine prevalence, with higher melanisation and lower gregarine prevalence in the warmer season. These findings highlight that seasonal climate fluctuations shape host-parasite interactions, providing valuable insights into patterns of disease dynamics and insect fitness. While brief periods of warming might strengthen host immune response, persistent or extreme climate change could upset host-parasite interactions by changing resources, humidity, and thermal tolerance limits for insects, which may lead to seasonal decrease in the ability to mount an immune response and impact host populations.

### Statement of diversity and inclusion

We believe, support, and practice equity, diversity, and inclusion in science and everywhere (Rößler et al. 2020). We come from different countries, nationalities, residency, ethnicity, and cultural backgrounds (Bangladesh, Austria, and Australia), and the neurodivergent community. We represent different career stages (Graduate student, early career researcher, and Professor). One or more of the authors self-identifies as a member of the LGBTQI + community and represents a religious minority in science.

## Supplementary Information

Below is the link to the electronic supplementary material.

Supplementary file1 (DOCX 12342 KB)

## Data Availability

All data for analysis are deposited in Figshare and can be accessed with the private link: https://figshare.com/s/acd36adb9201843828a2.

## Code availability

All codes used to analyse the data of this study are deposited in Figshare and can be accessed via following link: https://figshare.com/s/acd36adb9201843828a2.
