# Supplementary material for: Seasonal climatic variability shapes immune responses and infection risks in the common bluetail damselfly
Source: Oecologia. 2026 Mar 24;208(4):47. doi: 10.1007/s00442-026-05882-w (PMC13013159; doi:10.1007/s00442-026-05882-w)
Supplement: Supplementary file 1 — Supplementary file1 (DOCX 12342 KB) [file 442_2026_5882_MOESM1_ESM.docx]

**Seasonal climatic variability shapes immune responses and infection risks in the common bluetail damselfly**

Shatabdi Paul^1,6^, Md Tangigul Haque^1,6^, Marie E. Herberstein^1,3,4^, Md Kawsar Khan^1,2,5^

1. School of Natural Sciences, Macquarie University, NSW-2109, Australia
2. Department of Biology, Chemistry and Pharmacy, Freie Universität Berlin, Germany
3. Leibniz Institute for the Analysis of Biodiversity Change, Bonn, Germany
4. Department of Biology, University of Hamburg, Germany
5. Applied BioSciences, Macquarie University, NSW-2109, Australia
6. Department of Biochemistry and Molecular Biology, Primeasia University, Dhaka, Bangladesh

*Correspondence: Shatabdi Paul

School of Natural Sciences,

Macquarie University, NSW-2109, Australia

E-mail: [shatabdi.paul@students.mq.edu.au](mailto:shatabdi.paul@students.mq.edu.au)

**Supplementary information**

**Model description of main statistical analysis: Melanisation and gregarine prevalence between sexes**

At first, we investigated the influence of sex of damselflies on melanisation (Model 1 <- glm (melanisation ~ sex * season, data), and on gregarine prevalence (Model 2 <- glm (cbind (endo, endo_no_parasite) ~ sex * season, family = binomial, data)).

**Model description of main statistical analysis: Correlation of melanisation and gregarine prevalence with climatic factors between sexes**

We applied generalized linear models (GLMs) to identify the effect of climatic factors (monthly average temperature, rainfall, and humidity) on melanisation (Model 3 <- glm (melanisation ~ monthly average temperature + rainfall + relative humidity, data = female/ male); and on gregarine prevalence (Model 4 <- glm (gregarine prevalence ~ monthly average temperature + rainfall + relative humidity, data = female/ male)).

**Infection status and melanisation across seasons and the correlation of melanisation, monthly temperature, and their interaction with gregarine prevalence**

We also checked how infection status influenced melanisation in damselflies (Model 5 <- glm (melanisation ~ infection status, data)) and how both melanisation, and monthly temperature affected gregarine prevalence **(**Model 6 <- glm (gregarine prevalence ~ melanisation * monthly average temperature, data)).

**Mean differences of melanisation between gregarine-infected and non-infected damselflies across seasons**

We further applied the DurgaDiff function of the *Durga* R package to calculate mean differences of melanisation between gregarine-infected and non-infected damselflies across seasons (Khan and McLean, 2024). Please see supplementary results and figure (Fig. S1) below.

**Melanisation and gregarine prevalence across female developmental stages, seasons and climatic gradients**

We also applied generalized linear models (GLMs) to identify the influence of developmental stages of females on melanisation (Model 1* <- glm (melanisation ~ dev_stage + season, data = female)) and on gregarine prevalence (Model 2* <- glm (cbind (endo, endo_no_parasite) ~ dev_stage + season, family = binomial, data = female)). Again, we applied generalized linear models (GLMs) to identify the effect of climatic factors (monthly average temperature, rainfall, and humidity) on melanisation (Model 3* <- glm (melanisation ~ monthly average temperature + rainfall + relative humidity, data = mature female/ immature female); and on gregarine prevalence (Model 4* <- glm (gregarine prevalence ~ monthly average temperature + rainfall + relative humidity, data = mature female/ immature female)).

**Supplementary results**

**Melanisation across months**

Melanisation was highest in December (Female, December, GLM, estimate = 44.52 ± 5.2, t = 8.54, p < 0.0001; Male, December, GLM, estimate = 42.81 ± 5.05, t = 8.47, p < 0.0001).

**Melanisation and gregarine prevalence across seasons**

Melanisation was higher in summer than spring and lower in autumn for both sexes ((Female, Summer - Autumn, GLM, estimate = -26.53 ± 3.2, t = -8.28 p < 0.0001, Summer - Spring, GLM, estimate = -1.34 ± 2.86, t = -0.47, p = 0.63; Fig. 2b); (Male, Summer - Autumn, GLM, estimate = -20.72 ± 3.23, t = -6.41, p < 0.0001, Summer – Spring, GLM, estimate = -9.6 ± 3.03, t = -3.16 p = 0.001, Fig. 2c)).

Gregarine prevalence was relatively higher during spring and autumn than summer ((Female, Spring, GLM, estimate = 0.52 ± 0.87, t = 0.59, p = 0.55, Summer, GLM, estimate = -0.47 ± 0.78, t = -0.6, p = 0.54; Fig. 2d); Male, Spring, GLM, estimate = 0.55 ± 0.58, t = 0.95, p = 0.33, Summer, GLM, estimate = -1.16 ± 0.76, t = -1.52, p = 0.12; Fig. 2e)).

**Infection status and melanisation across seasons**

Melanisation was higher in infected females all over the seasons (Female, Autumn, non-infected-infected: -7.9, 95% CI [-26.71, 22.38]; Spring: -6.02, 95% CI [-13.16, 1.56]; Summer: -6.26, 95% CI [-20.36, 8.86]; Fig. S1a). However, infected males had stronger responses during spring than summer (Male, Autumn: 7.67, 95% CI [-15.12, 27.96]; Spring: -11.72, 95% CI [-23.34, -0.01]; Summer: -0.29, 95% CI [-20.27, 13.26]; Fig. S1b).

**Correlation of melanisation, monthly temperature, and their interaction with gregarine prevalence**

In females, we found no significant effect of melanisation (GLM, estimate = –0.006 ± 0.01, t = -0.33, p = 0.73), monthly average temperature (GLM, estimate = –0.08 ± 0.09, t = -0.94, p = 0.35), or their interaction (GLM, estimate = 0.0005 ± 0.0009, t = 0.56, p = 0.57) on gregarine prevalence. Although the coefficients suggested a slight decline in gregarine prevalence with increasing melanisation and monthly average temperature, the effects were not statistically significant.

Similarly in males, melanisation (GLM, estimate = -0.007 ± 0.02, t = -0.25, p = 0.8), monthly average temperature (GLM, estimate = -0.03 ± 0.12, t = -0.25, p = 0.8), and their interaction (GLM, estimate = 0.0003 ± 0.001, t = 0.24, p = 0.81) had no effects on gregarine prevalence.

**Melanisation and gregarine prevalence between mature and immature females across seasons**

Melanisation is higher in mature females than immature females (mean difference in greyscale value: 5.19, 95% CI [-0.98, 12.15]; (GLM, estimate = 11.19 ± 2.78, t = 4.01, p < 0.0001), Fig. S3a). Similarly, gregarine prevalence was higher in mature females than immature females (mean difference: 20.6744, 95% CI [-13.9216, 39.0404], Fig. S3d). Melanisation was higher in spring (average greyscale value: 102.4529 ± 15.29706; (Spring - Autumn, GLM, estimate = 27.16 ± 3.07, t = 8.85, p < 0.0001, Fig. S3b)), and in summer (average greyscale value: 101.6143 ± 14.06517; (Summer - Autumn, GLM, estimate = 29.23 ± 3.13, t = 9.33, p < 0.0001), Fig. S3b) and lower in autumn (75.19151 ± 16.55898) in mature female. For immature female, melanisation was higher in summer (average greyscale value: 96.1533 ± 13.20449; (Summer - Autumn, GLM, estimate = 41.09 ± 7.55, t =5 .43, p < 0.0001)), and in spring (average greyscale value: 87.73506 ± 15.26237, (Spring - Autumn, GLM, estimate = 32.67 ± 7.66, t = 4.26, p < 0.0001), and lower in autumn (average greyscale value: 55.06275 ± 8.04832; (Fig. S3c; Table S1)).

Gregarine prevalence was relatively higher during summer (93.33%), and autumn (88.23%) than spring (85%) in mature female (Fig. S3e; Table S1). In immature female, gregarine prevalence was higher in spring (100%), than in summer (54.54%) and in autumn (50%) (Fig. S3f; Table S1).

**Table S1:** Mean differences showing the variation in melanisation of mature and immature female *I. heterosticta* damselflies across seasons.

| **Sex** | **Response variable** | **Group difference** | **Mean difference** | **95% CI** |
| --- | --- | --- | --- | --- |
| Mature female | Melanisation | Summer - Autumn | 26.42 | [19.12, 33.26] |
|  |  | Summer - Spring | -0.83 | [-7.28, 4.94] |
| Immature female |  | Summer - Autumn | 41.09 | [30.99, 49.15] |
|  |  | Summer - Spring | 8.41 | [-0.71, 17.05] |
| Mature female | Gregarine prevalence | Spring - Autumn | 0.02 | [-0.20, 0.23] |
|  |  | Spring - Summer | - 0.13 | [-0.27, 0.04] |
| Immature female |  | Spring - Autumn | 0.5 | [0, 0.5] |
|  |  | Spring - Summer | 0.46 | [0.33, 0.57] |

**Correlation of melanisation and gregarine prevalence with climatic factors in mature and in immature female damselflies**

Melanisation was negatively correlated with rainfall (GLM, estimate = -0.09 ± 0.02, z = -3.282, p < 0.01, pseudo-R^2^ = 0.21; Fig. S4b) and with humidity (GLM, estimate = -0.60 ± 0.15, z = -3.98, p < 0.0001, pseudo-R^2^ = 0.21; Fig. S4c) in mature females, but had no significant association with temperature (GLM, estimate = 1.17 ± 0.60, z = 1.96, p = 0.05, pseudo-R^2^ = 0.21; Fig. S4a). Melanisation was again positively correlated with temperature (GLM, estimate = 3.12± 0.82, z = 3.79, p < 0.0001, pseudo-R^2^ = 0.34; Fig. S4a) and negatively correlated with humidity in immature females (GLM = -0.61± 0.27, z = -2.22, p < 0.05, pseudo-R^2^ = 0.34; Fig. S4c). However, there was no significant relationship between melanisation and rainfall in immature females (GLM, estimate = -0.02± 0.06, z = -0.42, p = 0.67, pseudo-R^2^ = 0.34; Fig. S4b).

Gregarine prevalence did not show any statistically significant correlation with monthly average temperature, rainfall and with humidity in mature females (temperature, GLM, estimate = -0.01 ± 0.01, z = -0.65, p = 0.52, Fig S3d, e, f; rainfall: GLM, estimate = 0.0007 ± 0.001, z = 0.72, p = 0.48; humidity: GLM, estimate = 0.007 ± 0.005, z = 1.54, p = 0.15; Fig. 3d) and in immature females, (temperature, GLM, estimate = -0.02 ± 0.039, z = -0.72, p = 0.48; rainfall: GLM, estimate = -0.005 ± 0.002, z = -1.75, p = 0.11; humidity: GLM, estimate = 0.003 ± 0.01, z = 0.258, p = 0.80; Fig S3d, e, f).

**Supplementary figures**

**
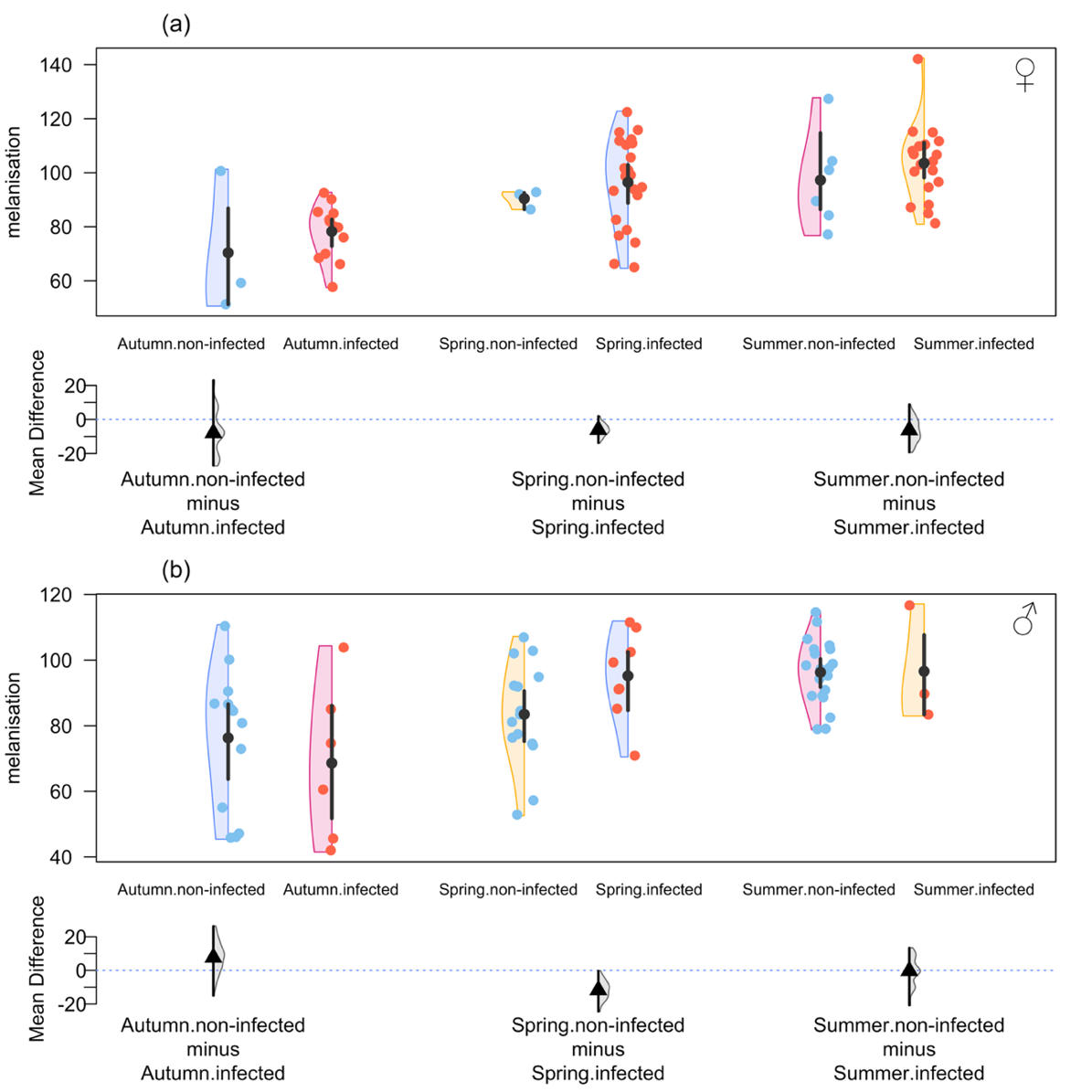
**

**Figure S1:** Melanisation response (Grey value) of gregarine non-infected and infected female and male *I. heterosticta* damselflies across seasons. Melanisation response of (a) gregarine non-infected and infected females and (b) males across seasons. In the upper panel of (a) and (b) black circle represents the mean, and the vertical bar represents the melanisation difference between non-infected and infected damselflies across seasons. In (a) and (b), coloured dots represent melanisations of non-infected and infected females and males, and each coloured circle represents a sampling event. In the lower panel, the triangle represents the mean difference, and the vertical line represents the 95% CI of the mean difference from 1000 bootstraps.

**
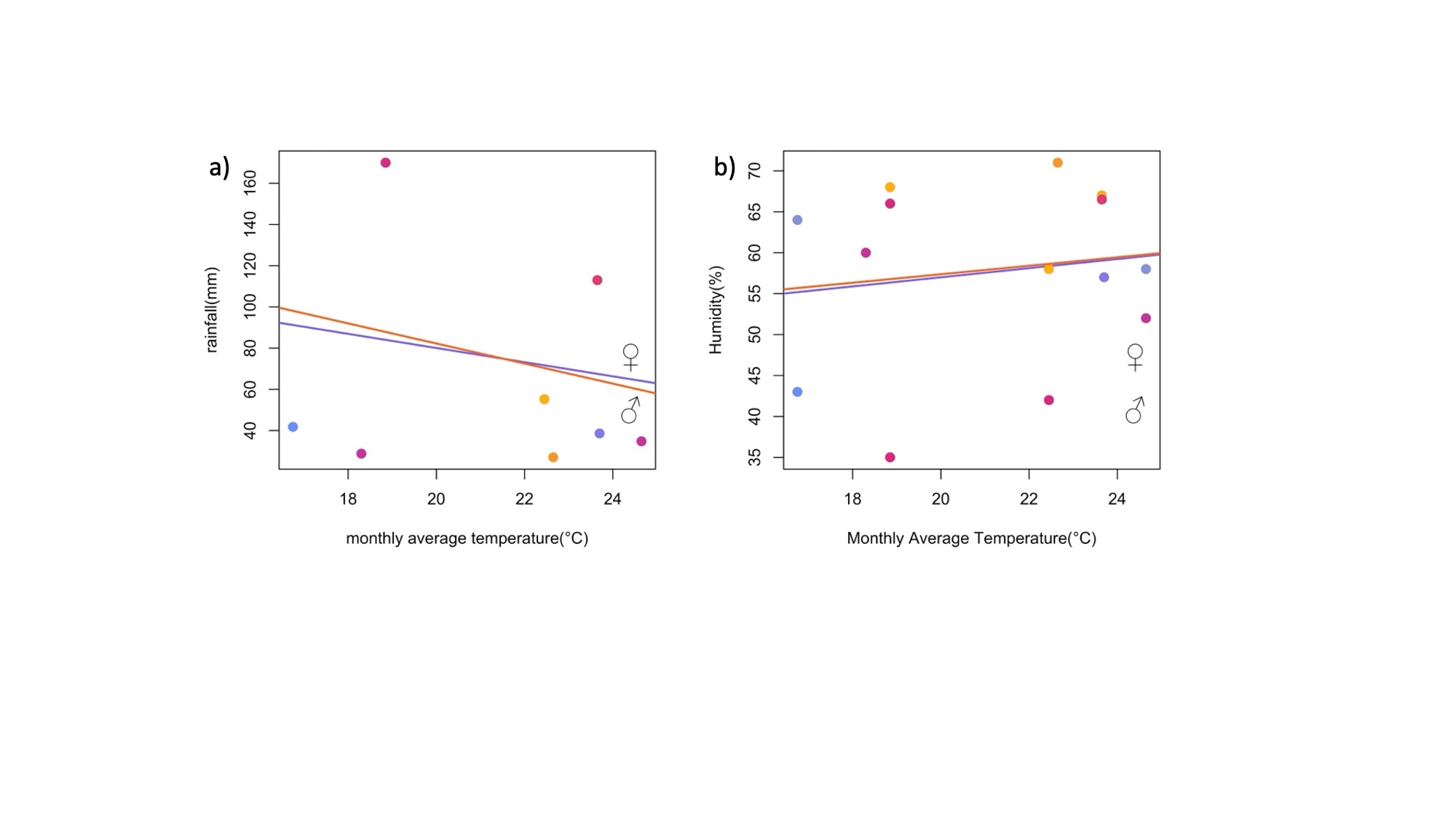
**

**Figure S2:** Correlation of monthly average temperature with rainfall and relative humidity across three seasons, represented by coloured dots in both sexes of *I. heterosticta* damselflies. Monthly temperatures were negatively correlated with rainfall but positively correlated with humidity for both sexes.


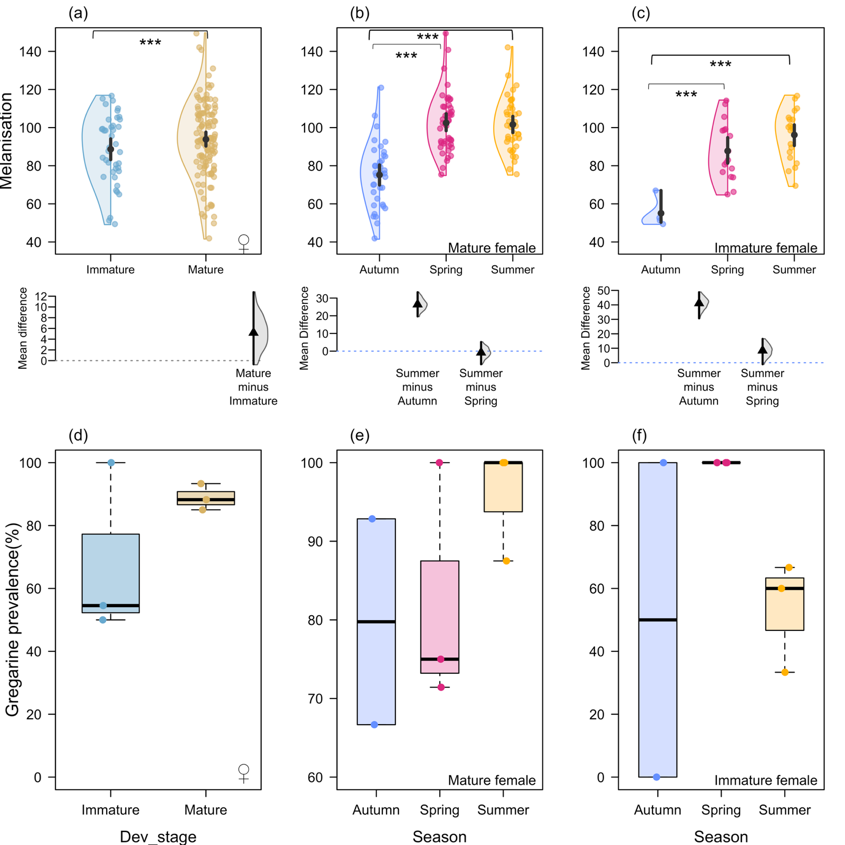


**Figure S3:** Melanisation response (greyscale value) and gregarine prevalence in *I. heterosticta* mature and immature female damselflies across seasons. Melanisation response and variation in gregarine prevalence (a), (d) between two developmental stages of females; across seasons (b), (e) in mature females; and (c), (e) in immature females. In the upper panel of plots (a-c), the black circle represents the mean, and the vertical bar represents confidence intervals (CI) of both mature and immature females across seasons. In (a), coloured dots represent melanisation, and each coloured circle in (b) and (c) represents a sampling event across seasons for mature and immature females, respectively. In the lower panel, the triangle represents the mean difference, vertical line represents the 95% CI of the mean difference from 1000 bootstraps. Boxplots (d-f), showing the difference in gregarine prevalence between mature and immature females, and across seasons, where bold lines indicate the median, and bottom and top borders depict the 25th and 75th percentiles. The error bars extend downward from the first quartile to the minimum and upward from the third quartile to the maximum data points. We used a subset of data (n = 10 from each sampling event) to determine the percentage of female damselflies infected.


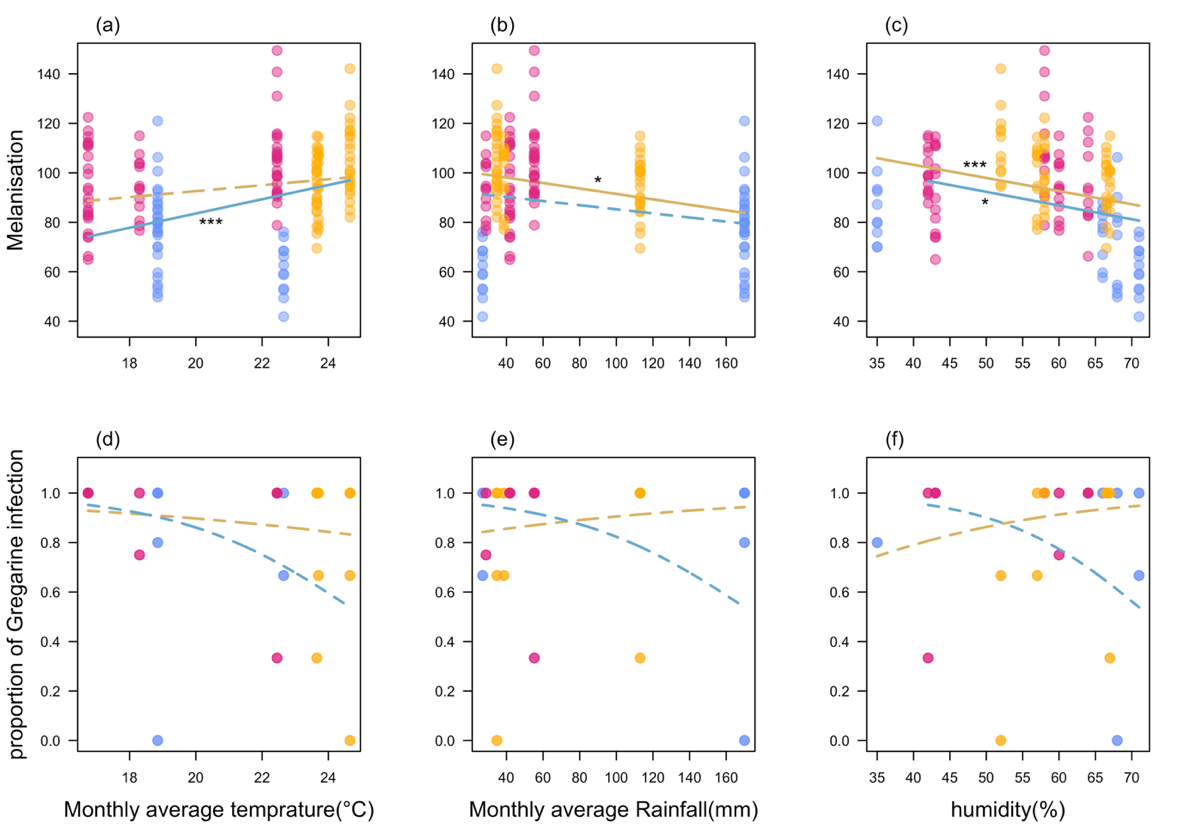


**Figure S4:** Correlation of melanisation response (greyscale value) and gregarine prevalence in *I. heterosticta* mature and immature female damselflies with climatic factors (monthly average temperature, rainfall, and humidity). Plots (a-c) and (d-f) show the correlation of melanisation and gregarine prevalence with climatic factors across three seasons in mature and immature females, respectively. Each circle represents a sampling event. The fitted lines represent the overall trend of the data points. Brown and blue coloured lines represent mature and immature female data respectively. Dashed lines represent non-significant statistical relationships. We used a subset of data (n = 5 from each sampling event) for calculating the effect of climatic factors on gregarine prevalence. Asterisks denote significance levels (*p < 0.05, *****p *<* 0.0001).
